# Mothers’ utilization and associated factors of preconception care in Africa, a systematic review and meta-analysis

**DOI:** 10.1371/journal.pone.0254935

**Published:** 2021-07-23

**Authors:** Tiwabwork Tekalign, Tesfanesh Lemma, Mulualem Silesh, Eyasu Alem Lake, Mistire Teshome, Tesfaye Yitna, Nefsu Awoke

**Affiliations:** 1 School of Nursing, College of Health Science and Medicine, Wolaita Sodo University, Wolaita Sodo, Ethiopia; 2 Department of Midwifery, College of Health Science and Medicine, Debre Berhan University, Debre Berhan, Ethiopia; Georgia Southern University, UNITED STATES

## Abstract

**Background:**

As the studies show, in every minute in the world, 380 women become pregnant and 190 face unplanned or unwanted pregnancies; 110 experience pregnancy-related complications, and one woman dies from a pregnancy-related cause. Preconception care is one of the proven strategies for the reduction in mortality and decreases the risk of adverse health effects for the woman, fetus, and neonate by optimizing maternal health services and improves woman’s health. Therefore, this study aimed to estimate the pooled prevalence of utilization of preconception of care and associated factors in Africa.

**Methods:**

Systematic search of published studies done on PubMed, EMBASE, MEDLINE, Cochrane, Scopus, Web of Science CINAHL, and manually on Google Scholar. This meta-analysis follows the Preferred Reporting Items for Systematic Reviews and Meta-Analyses (PRISMA) guidelines. The quality of studies was assessed by the modified Newcastle-Ottawa Scale (NOS). Meta-analysis was carried out using a random-effects method using the STATA^™^ Version 14 software.

**Result:**

From 249,301 obtained studies, 28 studies from 3 African regions involving 13067 women included in this Meta-analysis. The overall pooled prevalence of utilization of preconception care among pregnant women in Africa was found to be 18.72% (95% CI: 14.44, 23.00). Knowledge of preconception care (P = <0.001), preexisting medical condition (P = 0.045), and pregnancy intention (P = 0.016) were significantly associated with the utilization of preconception care.

**Conclusion:**

The results of this meta-analysis indicated, as one of best approaches to improve birth outcomes, the utilization of preconception care is significantly low among mothers in Africa. Therefore, health care organizations should work on strategies to improve preconception care utilization.

## Introduction

According to the World Health Organization (WHO), preconception care (PCC) is, the provision of biomedical, behavioral, and social health interventions to women and couples before conception occurs, to improve their health status, and mitigating behaviors, individual and environmental factors that could contribute to poor maternal and child health outcomes [1, 2]. This is done through risk identification, health education, and promotion, and initiation of evidence-based interventions in the period before conception. The use of PCC in high- and low-income countries aims to improve maternal pregnancy and neonatal outcomes both in the short and long term [3]. PCC also includes the detection and optimal control of specific medical conditions to optimize pregnancy-related outcomes for the woman and her offspring as well as implemented to prevent pregnancies that are unplanned, too early, or too close [4, 5].

Now a day promoting and enhancing women’s health before pregnancy has a favorable outcome and highly reduces pregnancy and childbirth related complications [6], and also Preconception care can make a useful contribution to reducing maternal and childhood mortality and morbidity, and to improving maternal and child health in both high- and low-income countries [1]. In 2015, 303, 000 women in the world died from pregnancy and childbirth-related problems [7]. In Ethiopia, the pregnancy-related mortality ratio was 412 per 100,000 live births and the lifetime risk of pregnancy-related death is 21 in 1000 women [8]. Most of these complications develop during pregnancy, exist before, and worsened during pregnancy, especially if not managed as part of the PCC [2]. There is growing evidence that preconception care may have an important role in preventing short and long-term adverse health consequences for women and their offspring [9].

Besides PCC is very crucial for women with underlying chronic diseases, according to global statistics, non-communicable diseases (NCDs) are the cause of more than 53% of diseases. Moreover, it is predicted that NCDs will be the cause of 73% of deaths worldwide and 80% of deaths in developing countries [10, 11]. Researchers reported in their studies that 17.5% and 32% of pregnant mothers who were referred to healthcare centers had received pre-pregnancy care. Similarly, the findings of the studies conducted by Asresu and Betra 18.2% and 29.7% of people seek pre-pregnancy care programs [12–15]. In another study conducted by Frey and Files in Mayo clinic [16] found that only 39% of the women received PCC from their primary care physicians compared to 98.6% who believed in its importance.

There has been an increasing burden of maternal, newborn, and child mortality globally. Worldwide, 400/100000 women of childbearing age die every year due to complications of pregnancy and childbirth and 7 million infants die each year between birth to 12 months [17]. According to statistics, every minute in the world, 380 women become pregnant and 190 faces unplanned or unwanted pregnancies; 110 experiences a pregnancy-related complication; 40 have an unsafe abortion; and one woman dies from a pregnancy-related cause. Implementation of evidence-based preconception interventions improves infant and maternal pregnancy outcomes [18]. This review will contribute to the integration of preconception care with other existing health programs, assignment of the task of pre-pregnancy health promotion to the healthcare workers, improvement or promotion of preconception services, engagement of the media, usage of healthcare information technology, maximizing demand for and uptake of preconception interventions, especially by adolescents.

## Objectives of the review

- To determine the prevalence of utilization of Preconception care in Africa
- To identify the associated factors of utilization Preconception care in Africa

## Methods and materials

### Study design and search strategy

We registered the protocol in PROSPERO (ID: CRD42020209551). This systematic review and meta-analysis was conducted under the guidelines of the Preferred Reporting Items for Systematic Reviews and Meta-analyses (PRISMA) statement [19, 20].

A three-step search strategy was utilized in this review. An initial limited search of PubMed was undertaken followed by the analysis of the text words contained in the title and abstract, and of the index terms used to describe the article. A second search was done by using all identified keywords and index terms across all included databases. Thirdly, the reference list of all identified reports and articles was searched for additional studies. Studies published in English language up to May 2021 were taken from EMBASE, MEDLINE, Cochrane, Scopus, Web of Science, CINAHL, and manually on Google Scholar. The search for unpublished studies included Google and institutional repositories. The search was performed using key terms such as preconception care, PCC, Pre-pregnancy care, prenatal care, folic acid, multi-vitamin, foliate supplement, folic acid intake, Iron–folic acid, IFA, Mother, reproductive age group, pregnant women, utilization, and uptake.

### Study selection and eligibility criteria

- Participants in the studies should be mothers.
- Both published and unpublished studies conducted in Africa were included.
- Studies that reported the prevalence of utilization of preconception care among mothers regardless of study design

### Study extraction and quality appraisal

The data were extracted by three independent authors (TT, MT, and T.L) using a data extraction format prepared in a Microsoft Excel 2010 spreadsheet. The extracted data were: the first author’s name, publication year, country, design, sample size, sampling method, utilization of preconception care, and associated factors with their odds ratio. The quality of each study was assessed using the modified Newcastle-Ottawa Scale (NOS) for cross-sectional studies [21, 22] Studies were included with a score of 5 and more on the NOS [23]. The quality of each study was evaluated independently by four authors (TT, NA, MT, and T.L) and ay disagreements were resolved by discussion and consensus.

### Publication bias and heterogeneity

To assess the existence of publication bias, funnel plots were used and Egger’s test was computed. A p-value< 0.05 was used to declare the statistical significance of publication bias. I2 test statistics were used to check the heterogeneity of studies. I^2^ test statistics of < 50, 50–75% and > 75% was declared as low, moderate and high heterogeneity respectively [24].

### Outcome measure

The primary outcome of this review was the prevalence of utilization of preconception care. The second outcome of this review was the associated factors of preconception care utilization. The only factor identified as a significant factor in the two and above studies was included in this review and meta-analysis.

### Data synthesis and analysis

STATA^™^ Version 14 software was used to conduct the analysis. The heterogeneity test was conducted by using I-squared (I^2^) statistics. The pooled prevalence of utilization of preconception care was carried out using a random-effects (DerSimonian and Laird) method. To minimize the potential random variations between studies; the sources of heterogeneity were analyzed using subgroup analysis, and meta-regression. A sensitivity analysis was also conducted.

## Results

### Study selection

**Fig 1:**
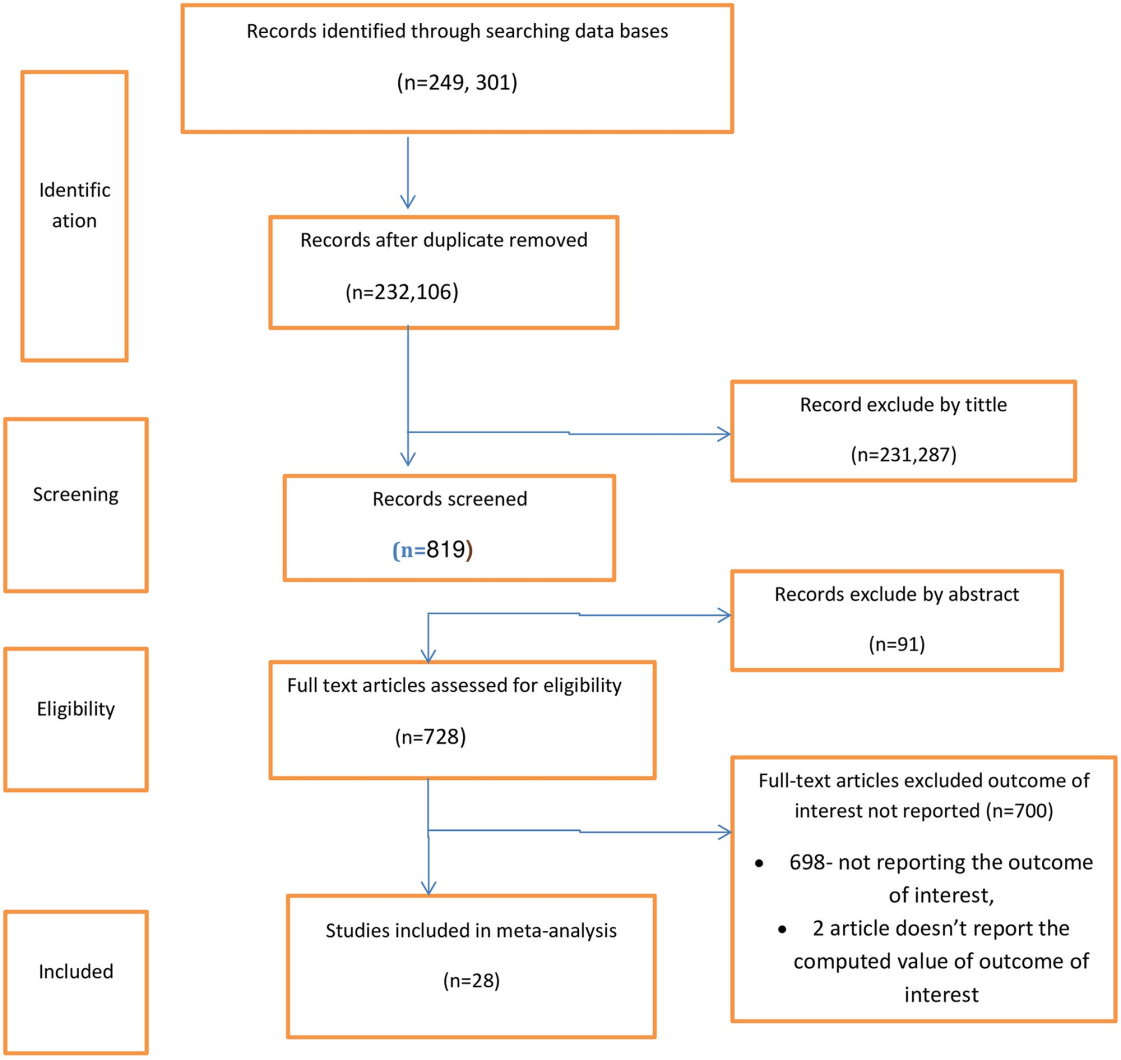
PRISMA flow diagram of study selection.

Initially, a total of 249,301 studies were retrieved from the databases and manual searching. From this, 17195 duplicates were found and removed. The remaining 232,106 articles were screened by their title and abstract and 231378 irrelevant studies were removed. 728 full-text articles were assessed for eligibility and 700 of them were excluded due to not reporting the outcome of interest, which doesn’t report the computed value of the outcome of interest. Finally, a total of 28 studies was fulfilled the inclusion criteria and enrolled in the study (Fig 1).

### Study characteristics

The 28 studies [25–51] included 13067 participants. All of the included studies were cross-sectional studies and the sample size ranged from 50 [31] to 1331 [43]. Most studies were conducted in Ethiopia. Among the included studies, utilization of preconception care among mothers were ranged from 2.5 [40] to 86.8 [31] (Table 1).

**Table 1:** Characteristics of the included studies in the systematic review and meta-analysis.

Authors Name	Publication Year	Study setting	Country	Study design	sample	prevalence%(95%CI)
Goshu, Y. A., et al	2018	Adet town	Ethiopia	Cross-sectional	229	9.6(5.78–13.41)
Asresu, T. T., et al	2019	Mekelle City	Ethiopia	Cross-sectional	561	18.2(15.0–21.39)
Demisse, T. L., et al	2019	Debre Birhan Town	Ethiopia	Cross-sectional	410	13.4(10.10–16.69)
Okemo, J.,	2020	Aga Khan University Hospital & Maragua Level Four Hospital	Kenya	Cross-sectional	194	25.8(19.64–31.95)
Fekene, D. B., et al	2020	west shoa	Ethiopia	Cross-sectional	669	14.5(11.83–17.16)
Metasebia Getachew	Unpublished	Debre Berhan	Ethiopia	Cross-sectional	413	16.5(12.92–20.07)
Olowokere, A.E et al	2015	Osun State	Nigeria	Cross-sectional	375	34.1(29.30–38.89)
Akinajo, O. R et al	2020	Lagos	Nigeria	Cross-sectional	50	86.8(77.41–96.18)
Gezahegn, A. (2016)	Unpublished	west Shoa Zon	Ethiopia	Cross-sectional	634	38.2(34.41–41.98)
Napoleon N. Ekem, et al	2018	teaching hospital Abakaliki	Nigeria	Cross-sectional	453	10.3(7.50–13.09)
Adeyemo, A. A., & Bello, O. O.	2021	University College Hospital, Ibadan	Nigeria	Cross-sectional	414	18.8(15.03–22.56)
Taddese F.	Unpublished	St. Paul’s Millennium Medical College	Ethiopia	Cross-sectional	280	18.1(13.59–22.6)
Setegn M.	2021	Mizan Aman	Ethiopia	Cross-sectional	605	16.2(13.26–19.13)
Teshome F,	2021	Manna District	Ethiopia	Cross-sectional	623	6.3(4.39–8.20)
Lawal TA, Adeleye AO.	2014	Ibadan	Nigeria	Cross-sectional	602	2.5(1.25–3.74)
Alsammani MA, et al	2017	Sudan	Sudan	Cross-sectional	1000	3.2(2.10–4.29)
Boakye-Yiadom AK, et al	2020	Tamale west hospital	Ghana	Cross-sectional	200	15(10.05–19.94)
Ahmed K, et al	2015	Sudan	Sudan	Cross-sectional	100	40(30.39–49.6)
Ezegwui HU,	2008	Nigeria	Nigeria	Cross-sectional	1331	47.7(45.01–50.38)
Al Darzi W, et al	2014	Ain Shams University Hospital	Egypt	Cross-sectional	660	8.8(6.63–10.96)
Dessie MA, et al	2017	Adama hospital medical college	Ethiopia	Cross-sectional	417	3.5(1.73–5.26)
Okon UA, et al	2020	Benue State	Nigeria	Cross-sectional	586	27.6(23.98–31.21)
Abdulmalek LJ.	2017	Benghazi	Libya	Cross-sectional	131	6(1.93–10.06)
Senoga I.	Unpublished	KCCA health centers, Kampala.	Uganda	Cross-sectional	423	16.5(12.96–20.03)
Adebo OO, et al	2017	Nigeria	Nigeria	Cross-sectional	300	3(1.06–4.93)
Anzaku AS.	2013	Jos	Nigeria	Cross-sectional	543	7.4(5.19–9.60)
C A. ENUKU, & FO. Adeyemo	2019	delta state	Nigeria	Cross-sectional	273	24.2(19.11–29.28)
Habte A, et al	2020	Southern state	Ethiopia	Cross-sectional	591	6.4(4.42–8.37)

### Utilization of preconception care among mothers

**Fig 2:**
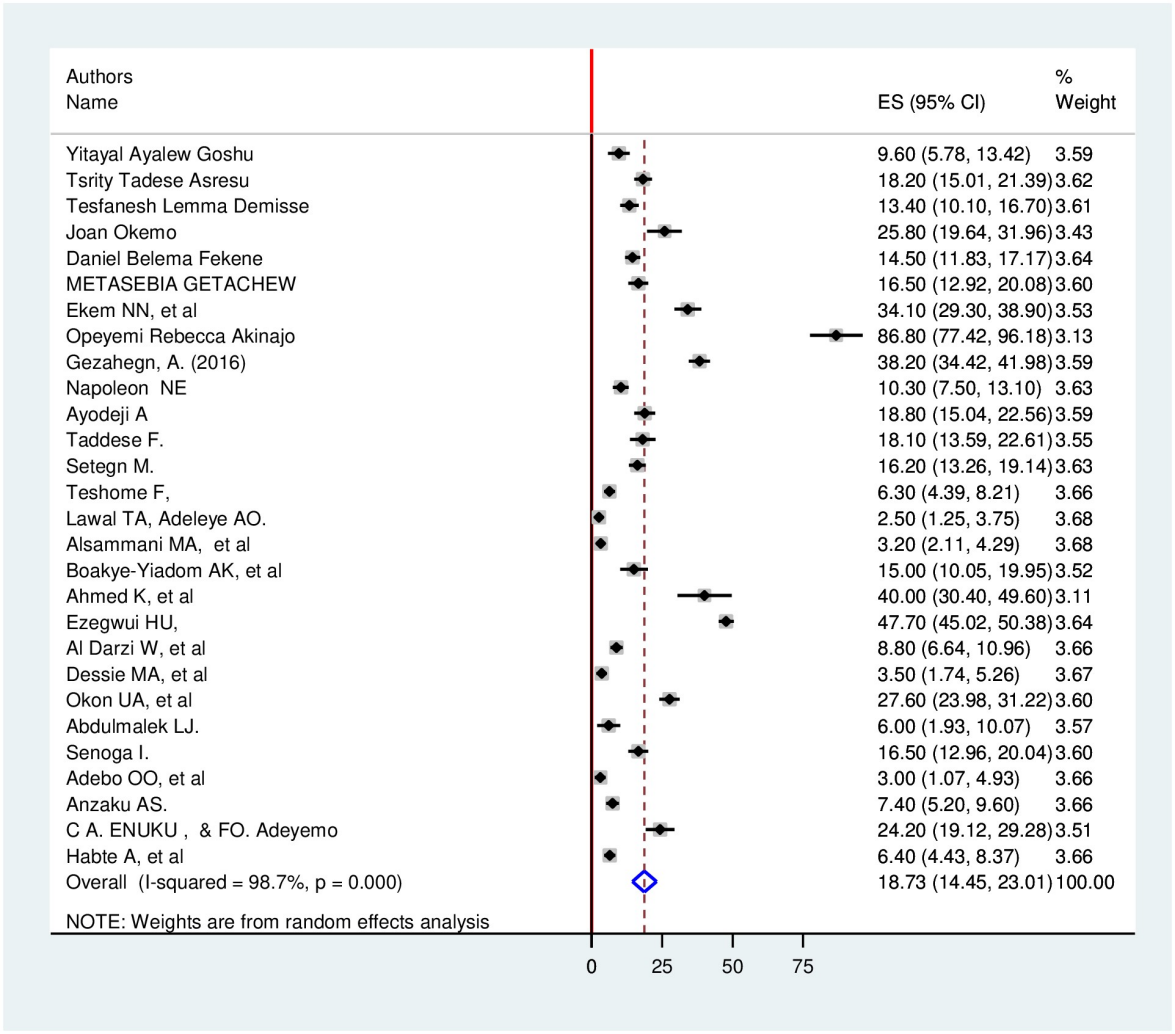
Forest plot showing pooled prevalence of utilization of preconception care among women in Africa.

By including the twenty-eight published research articles we had estimated the pooled prevalence utilization of preconception care among mothers in Africa. Accordingly, the overall estimated pooled prevalence of utilization of preconception care among mothers with a random-effects model was 18.72% (95% CI: 14.44, 23.00) with a heterogeneity index (I^2^) of 98.7% (p = 0.000) (Fig 2).

### Subgroup analysis

**Fig 3:**
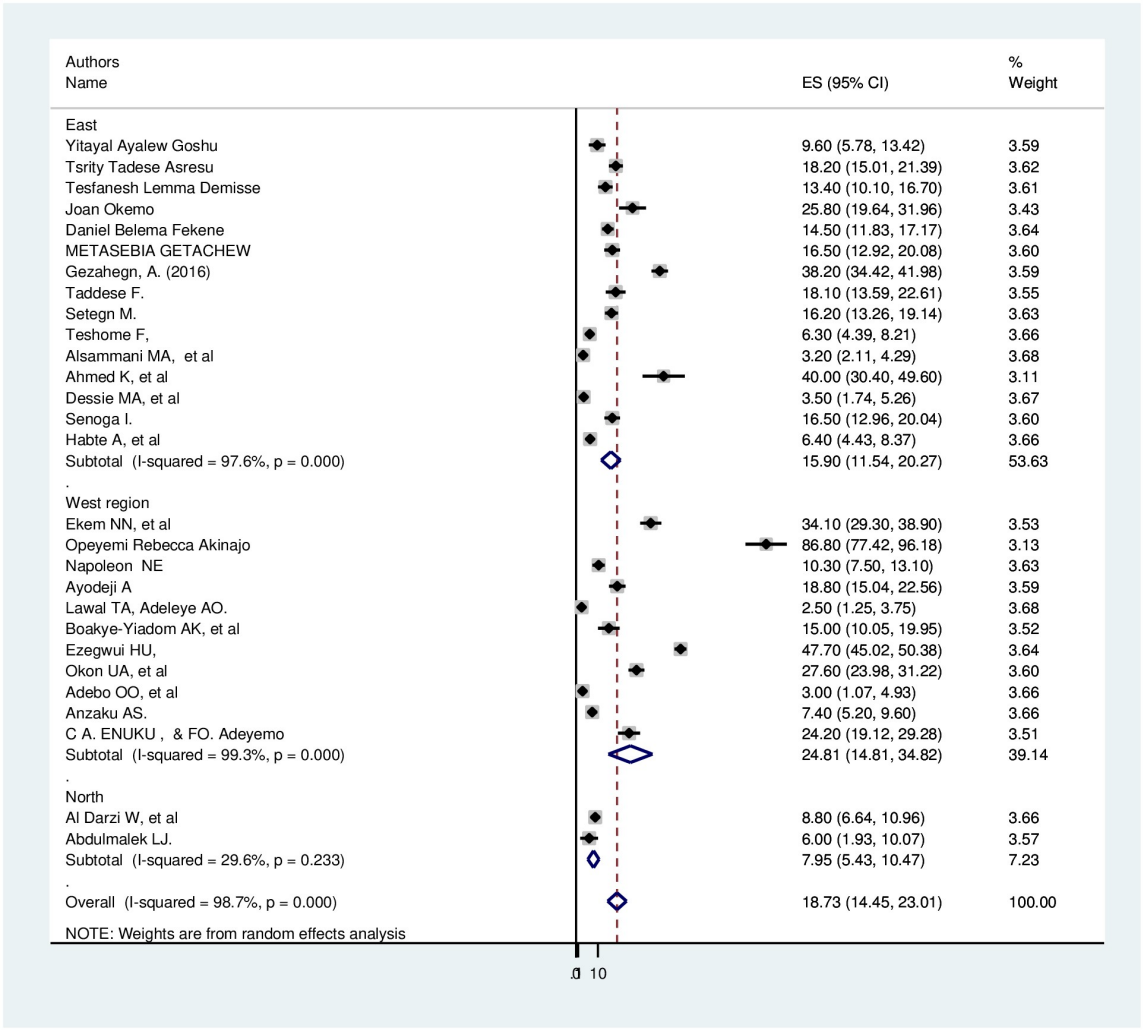
Subgroup analysis of utilization of preconception care among mother by country in Africa.

Subgroup analyses revealed a marked variation across regions. Based on the subgroup analysis result, the highest (24.81%; 95% CI: 14.80, 34.82), I^2^ = 99.3%) seen in western region and the lowest (15.90%; 95% CI: 11.54, 20.26), I^2^ = 97.6%) seen in eastern region (Fig 3).

### Heterogeneity and publication bias

**Fig 4:**
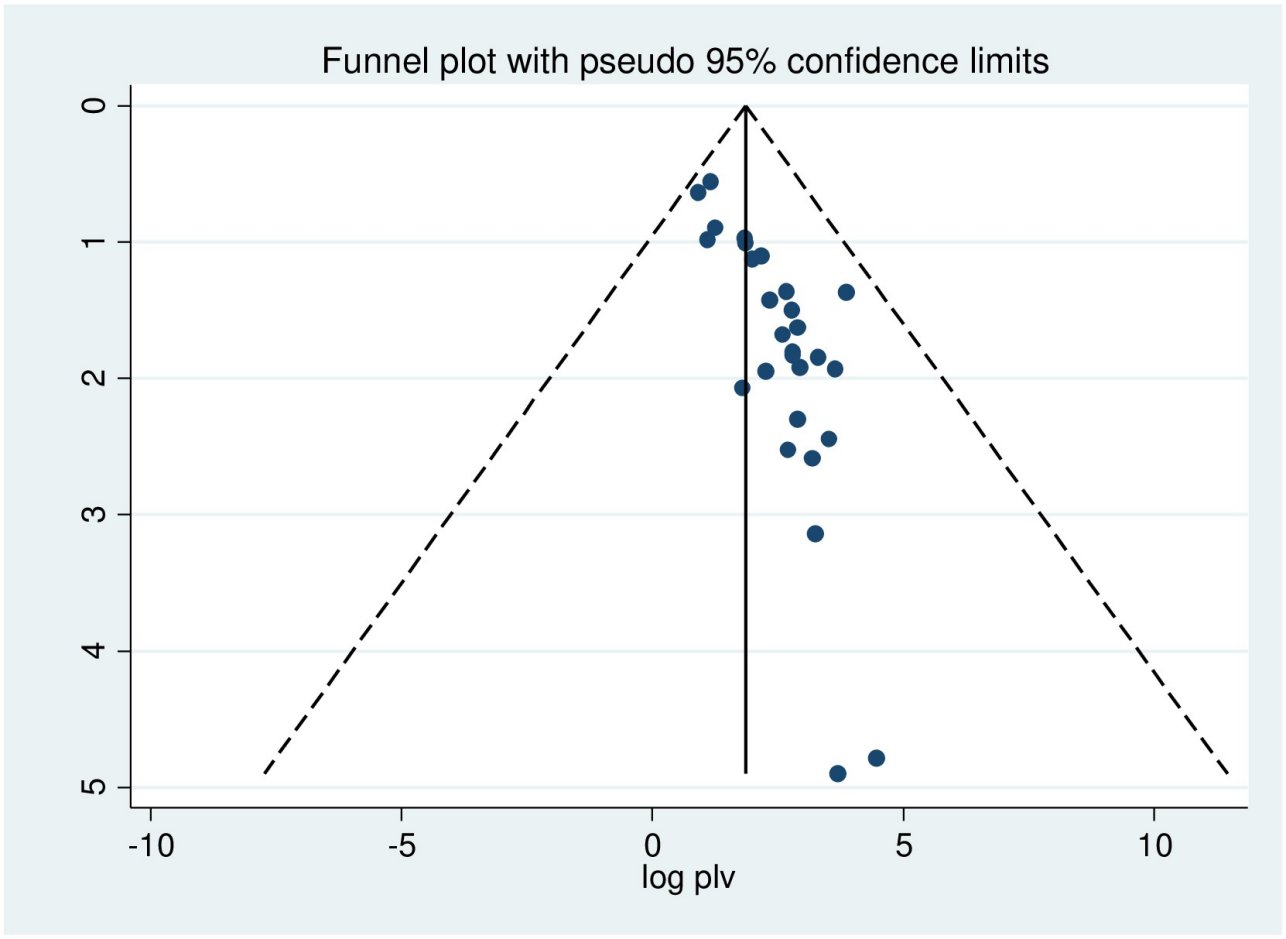
Funnel plot to test the publication bias in 28 studies with 95% confidence limits.

Meta-regression was conducted to identify the source of heterogeneity using sample size as a covariate (Table 2). It was indicated that there is no effect of sample size on heterogeneity between studies. The presence of publication bias was checked using the Egger’s test, and graphical by Funnel plot, the result egger’s test was found significant (p<0.000), as a result to estimating the number of missing studies that might exist in a meta-analysis we conducted Duval and Tweedie’s trim and fill analysis, but is not significant. Also, visual inspection of the funnel plot indicated asymmetrical distribution showing publication bias (Fig 4).

**Table 2:** Meta-regression analysis of factors affecting between-study heterogeneity.

Heterogeneity source	Coefficients	Std. Err.	P-value
Sample size	-0.0031257	.0052047	0.553

### Sensitivity analysis

**Fig 5:**
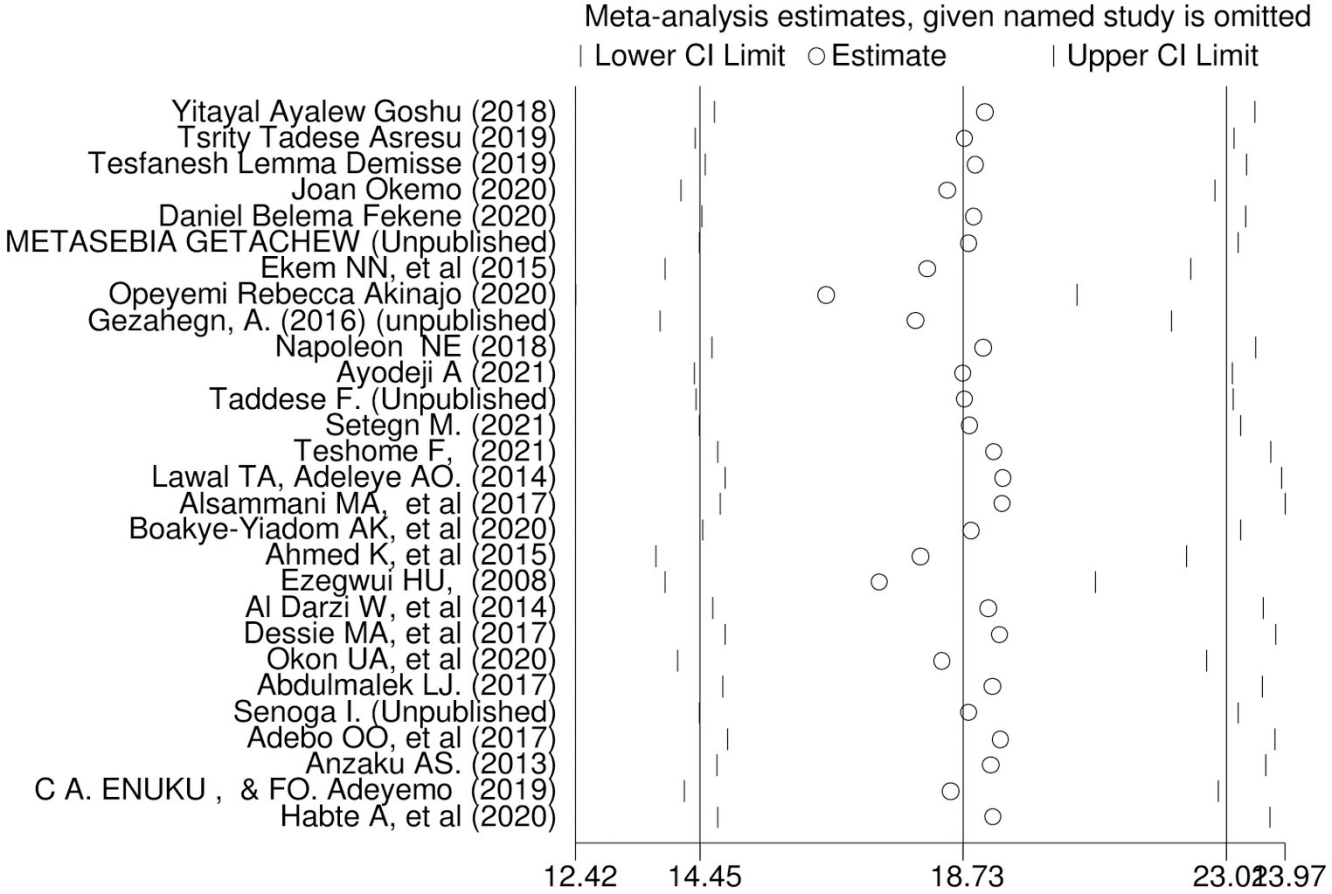
Sensitivity analysis of pooled prevalence for each study being removed one at a time.

Sensitivity analysis was done by removing studies step by step to evaluate the effect of a single study on the overall effect estimate. The result indicated removing a single study did not have a significant influence on pooled prevalence (Fig 5).

### Factors associated with utilization of preconception care

Six variables were extracted to identify factors affecting the utilization of preconception care among women. Of these, three variables (knowledge, Pre-existing medical condition, and pregnancy intention) were found to be significantly associated with utilization of preconception care (Table 3).

**Table 3:** Factors associated with utilization of preconception care among women in Africa.

Determinants	Comparison	Number of studies	Sample size	OR(95%CI)	P- value	I^2^ (%)	Heterogeneity test (p value)
Knowledge	Poor Vs Good Knowledge	7	3581	0.61(0.51–0.74)	< 0.001	97.5	< 0.001
Educational status	No formal education Vs Primary school and above	7	3475	0.68(0.44–1.05)	0.084	80.0	0.007
Marital status	Single Vs others	3	1103	0.68(0.44–1.05)	0.084	80.0	0.007
Pre-existing medical condition	Yes vs No	4	1615	0.71(0.51–0.99)	0.042	96.7	< 0.001
Adverse birth outcome	Yes vs No	2	845	1.15(0.81–1.64)	0.416	98.7	< 0.001
Pregnancy intension	Yes vs No	3	1247	2.47(1.74–3.52)	0.000	75.9	0.016

Mothers having poor knowledge of preconception care were 39% less likely to utilize the care than those having good knowledge (OR: 0.61(95% CI 0.51–0.74), p = 0.000, I^2^: 97.5%, the heterogeneity test (p< 0.001). Those mothers who had pre-existing medical condition were 29% less likely to utilize preconception care than their counterparts (OR: 0.71(95% CI 0.51–0.99), P = 0.045, I^2^: 96.7%, the heterogeneity test (p< 0.001).

Mothers who had pregnancy intention were 2.5 times more likely to utilize preconception care than those who hadn’t have an intention (OR: 2.47(95% CI 1.74–3.52), P = 0.000, I^2^: 75.9%, the heterogeneity test (p = 0.016).

## Discussion

Many medical conditions, personal behaviors, psychosocial risks, and environmental exposures associated with negative pregnancy outcomes can be identified and modified before conception through clinical interventions. For certain conditions, opportunities for preventive interventions occur only before conception, this is by preconception care. PCC is one way believed to improve pregnancy outcomes and is considered important by health care workers and the general population [52, 53].

A systematic review of tools to assess the quality of observational studies examining incidence or prevalence concluded that there is no consensus exists as to which individual criteria should be assessed to establish methodological quality [54].

The Cochrane Collaboration advises assessing the risk of bias on a subjective basis using domain-based evaluation [55], so we used the Newcastle-Ottawa Quality Assessment Scale (adapted for cross-sectional studies) [22] and authors independently reviewed with minimal disagreement between reviewers.

According to this systematic review and meta-analysis, the estimated pooled prevalence of utilization of preconception care among mothers was 18.72% (95% CI: 14.44, 23.00). It was believed that preconception care helps to fill the gap in the existing continuum of maternal and child healthcare [56]. Different randomized controlled trials also showed that one of PCC component folate supplementation (alone, or in combination with other vitamins and minerals) reduces the prevalence of neural tube defect [57–59].

Every woman of reproductive age who is capable of becoming pregnant is a candidate for preconception care, regardless of whether she is planning to conceive [60]. But PCC implementation is in the infant stage in low and middle-income countries [61].

Based on the subgroup analysis result, the highest (24.81%; 95% CI: 14.80, 34.82), I2 = 99.3%) seen in western region and the lowest (15.90%; 95% CI: 11.54, 20.26), I2 = 97.6%) seen in eastern region. The difference might be because of the difference in sample size, socioeconomic status of the countries, and the number of included studies in this meta-analysis.

Among the extracted factors preconception care knowledge, pre-existing medical condition, and pregnancy intention were found to be significantly associated with utilization of preconception care.

Seven studies revealed that having adequate knowledge about PCC was strongly associated with the utilization of PCC. Mothers having poor knowledge of preconception care were 39% less likely to utilize the care than those having good knowledge (OR: 0.61(95% CI 0.51–0.74); this is consistent with a systematic review conducted in Ethiopia [62]. knowing enhances the utilization of any health-related service.

Having pre-existing medical conditions has an impact on the utilization of PCC. In this study, those mothers who had pre-existing medical conditions were 29% less likely to utilize preconception care than their counterparts (OR: 0.71(95% CI 0.51–0.99). this might be explained as those having pre-existing medical conditions entirely worry about their medical condition than using preconception care service. Also, a study conducted in Nigeria showed that none of the participants with pre-existing medical conditions had awareness of PCC [63].

Pregnancy intention is one of the means which facilitates using of preconception care service. According to this study, mothers who had pregnancy intention were 2.5 times more likely to utilize preconception care than those who hadn’t have the intention (OR: 2.47(95% CI 1.74–3.52). If women had the intention to have a healthy baby, the probability of using PCC will increase.

## Conclusion

The results of this meta-analysis indicated as one of best approaches to improve birth outcomes, the utilization of preconception care is significantly low among mothers in Africa. Therefore, health care organizations should work on strategies to improve preconception care utilization.

## Limitation of the study

This systematic review and meta-analysis presented the prevalence of preconception care utilization in Africa; it might have faced the following limitations. First, the lack of studies from southern and middle Africa may affect the generalizability of the finding to Africa. Secondly, due to the presence of significant heterogeneity and presence of publication bias, the result should be interpreted cautiously. Finally, we have faced difficulties in comparing our findings due to the lack of regional and worldwide systematic reviews and meta-analysis.

## Supporting information

S1 Checklist PRISMA 2009 checklist. (DOC)

S1 Data Raw data. (XLSX)

## Abbreviations

CI: Confidence Interval
NCD: Non Communicable Disease
NOS: Newcastle Ottawa Scale
OR: Odds Ratio
PCC: Preconception care
PRISMA: Preferred Reporting Items for Systematic Reviews and Meta-Analyses
WHO: World Health Organization

## References

[1] World Health Organization. Meeting to develop a global consensus on preconception care to reduce maternal and childhood mortality and morbidity: World Health Organization Headquarters, Geneva, 6–7 February 2012: meeting report.

[2] Say L, Chou D, Gemmill A, Tunçalp Ö, Moller AB, Daniels J, et al. Global causes of maternal death: a WHO systematic analysis. The Lancet global health. 2014 Jun 1;2(6):e323–33. doi: 10.1016/S2214-109X(14)70227-X 25103301

[3] Jack BW, Atrash H, Coonrod DV, Moos MK, O’Donnell J, Johnson K. The clinical content of preconception care: an overview and preparation of this supplement. American journal of obstetrics and gynecology. 2008 Dec 1;199(6):S266–79. doi: 10.1016/j.ajog.2008.07.067 19081421

[4] Dunlop AL, Jack BW, Bottalico JN, Lu MC, James A, Shellhaas CS, et al. The clinical content of preconception care: women with chronic medical conditions. American journal of obstetrics and gynecology. 2008 Dec 1;199(6):S310–27. doi: 10.1016/j.ajog.2008.08.031 19081425

[5] World Health Organization. Pre-conception care: maximizing the gains for maternal and child health 2013. http://www.who.int/maternal_child_adolescent/documents/preconception_care_policy_brief.pdf.

[6] Elsinga J, de Jong-Potjer LC, van der Pal-de KM, le Cessie S, Assendelft WJ, Buitendijk SE. The effect of preconception counselling on lifestyle and other behaviour before and during pregnancy. Women’s Health Issues. 2008 Nov 1;18(6):S117–25. 19059545 10.1016/j.whi.2008.09.003

[7] Alkema L, Chou D, Hogan D, Zhang S, Moller AB, Gemmill A, et al. Global, regional, and national levels and trends in maternal mortality between 1990 and 2015, with scenario-based projections to 2030: a systematic analysis by the UN Maternal Mortality Estimation Inter-Agency Group. The Lancet. 2016 Jan 30;387(10017):462–74. 10.1016/S0140-6736(15)00838-7 PMC5515236 26584737

[8] Central Statistical Agency (CSA) [Ethiopia] and ICF. 2016. Ethiopia Demographic and Health Survey 2016: Key Indicators Report. Addis Ababa, Ethiopia, and Rockville, Maryland, USA. CSA and ICF.

[9] Steel A, Lucke J, Adams J. The prevalence and nature of the use of preconception services by women with chronic health conditions: an integrative review. BMC women’s health. 2015 Dec;15(1):1–2. doi: 10.1186/s12905-015-0165-6 25783639 PMC4338627

[10] Ghaffari Sardasht F, Jahani Shourab N, Jafarnejad F, Esmaily H. The frequency of risk factors associated with pregnancy among women seeking planned pregnancy. Journal of Midwifery and Reproductive Health. 2017 Jul 1;5(3):942–9.

[11] Lassi ZS, Imam AM, Dean SV, Bhutta ZA. Preconception care: screening and management of chronic disease and promoting psychological health. Reproductive Health. 2014 Dec;11(3):1–20. doi: 10.1186/1742-4755-11-S3-S5 25415675 PMC4196564

[12] Jahani Shourab N, Ghaffari Sardasht F, Jafarnejad F, Esmaili H. Assessment of prenatal care process based on donabedian model in Mashhad health centers. The Iranian Journal of Obstetrics, Gynecology and Infertility. 2013;16(53):7–17.

[13] Ebadi T, Narjes Sadat B, Bayrami R, Mehrbakhsh Z. Preconception care patterns and some related factors in pregnant women in Gorgan in 2017. JRDNM 2019 Nov 10;16(2):30–40.

[14] Asresu TT, Hailu D, Girmay B, Abrha MW, Weldearegay HG. Mothers’ utilization and associated factors in preconception care in northern Ethiopia: A community based cross sectional study. BMC pregnancy and childbirth. 2019 Dec;19(1):1–7. 31601190 10.1186/s12884-019-2478-1 PMC6787988

[15] Batra P, Higgins C, Chao SM. Previous adverse infant outcomes as predictors of preconception care use: an analysis of the 2010 and 2012 Los Angeles mommy and baby (LAMB) surveys. Maternal and child health journal. 2016 Jun;20(6):1170–7. doi: 10.1007/s10995-015-1904-x 26679708

[16] Frey KA, Files JA. Preconception healthcare: what women know and believe. Maternal and child health journal. 2006 Sep 1;10(1):73–7. doi: 10.1007/s10995-006-0110-2 16775757 PMC1592249

[17] Nepali G, Sapkota SD. Knowledge and practice regarding preconception care among antenatal mothers. International Journal of Perceptions in Public Health. 2017;1(4):224–7.

[18] Atrash HK, Johnson K, Adams MM, Cordero JF, Howse J. Preconception care for improving perinatal outcomes: the time to act. Maternal and child health journal. 2006 Sep 1;10(1):3–11. 10.1007/s10995-006-0100-4 PMC1592246 16773452

[19] Moher D, Liberati A, Tetzlaff J, Altman DG. Research methodes and reporting. Bmj. 2009 Aug 8;8:332–6.

[20] Liberati A, Altman DG, Tetzlaff J, Mulrow C, Gøtzsche PC, Ioannidis JP, et al. The PRISMA statement for reporting systematic reviews and meta-analyses of studies that evaluate health care interventions: explanation and elaboration. Journal of clinical epidemiology. 2009 Oct 1;62(10):e1–34. doi: 10.1016/j.jclinepi.2009.06.006 19631507

[21] Modesti PA, Reboldi G, Cappuccio FP, Agyemang C, Remuzzi G, Rapi S, et al. Panethnic differences in blood pressure in Europe: a systematic review and meta-analysis. PloS one. 2016 Jan 25;11(1):e0147601. doi: 10.1371/journal.pone.0147601 26808317 PMC4725677

[22] Modesti P. A., Reboldi G., & Cappuccio FP., Newcastle-Ottawa Quality Assessment Scale (adapted for cross sectional studies). PLoS One 2016

[23] Herzog R, Álvarez-Pasquin MJ, Díaz C, Del Barrio JL, Estrada JM, Gil Á. Are healthcare workers’ intentions to vaccinate related to their knowledge, beliefs and attitudes? A systematic review. BMC public health. 2013 Dec;13(1):1–7. doi: 10.1186/1471-2458-13-154 23421987 PMC3602084

[24] Higgins JP, Thompson SG, Deeks JJ, Altman DG. Measuring inconsistency in meta-analyses. Bmj. 2003 Sep 4;327(7414):557–60. doi: 10.1136/bmj.327.7414.557 12958120 PMC192859

[25] Goshu YA, Liyeh TM, Ayele AS. Preconception care utilization and its associated factors among pregnant women in Adet, North-Western Ethiopia (Implication of Reproductive Health). J Women’s Health Care. 2018;7(445):2167–0420.

[26] Gezahegn, A. (2016). Assessment Knowledge and Experience of Preconception Care among Pregnant Mothers attending Antenatal Care in West Shoa Zone Public Health Centers, 2016 (Doctoral dissertation, Addis Ababa University).

[27] Fekene DB, Woldeyes BS, Erena MM, Demisse GA. Knowledge, uptake of preconception care and associated factors among reproductive age group women in West Shewa zone, Ethiopia, 2018. BMC women’s health. 2020 Dec;20(1):1–8. 32075638 10.1186/s12905-020-00900-2 PMC7029592

[28] Demisse TL, Aliyu SA, Kitila SB, Tafesse TT, Gelaw KA, Zerihun MS. Utilization of preconception care and associated factors among reproductive age group women in Debre Birhan town, North Shewa, Ethiopia. Reproductive health. 2019 Dec;16(1):1–0. 31277717 10.1186/s12978-019-0758-x PMC6612124

[29] METASEBIA, G. (2020). Utilization of preconception care and associated factors among HIV positive women attending art clinics in north Shoa zone governmental hospitals, 2020 (Doctoral dissertation).

[30] Akinajo OR, Osanyin GE, Okojie OE. Preconception care: assessing the level of awareness, knowledge and practice amongst pregnant women in a tertiary facility. Journal of Clinical Sciences. 2019 Jul 1;16(3):87.

[31] Olowokere AE, Komolafe A, Owofadeju C. Awareness, knowledge and uptake of preconception care among women in Ife Central Local Government Area of Osun State, Nigeria. Journal of Community Medicine and Primary Health Care. 2015;27(2):83–92.

[32] Okemo J, Temmerman M, Mwaniki M, Kamya D. Preconception Care among Pregnant Women in an Urban and a Rural Health Facility in Kenya: A Quantitative Study. International journal of environmental research and public health. 2020 Jan;17(20):7430. 10.3390/ijerph17207430 PMC7601657 33065989

[33] Ekem NN, Lawani LO, Onoh RC, Iyoke CA, Ajah LO, Onwe EO, et al. Utilisation of preconception care services and determinants of poor uptake among a cohort of women in Abakaliki Southeast Nigeria. Journal of Obstetrics and Gynaecology. 2018 Aug 18;38(6):739–44. doi: 10.1080/01443615.2017.1405922 29526148

[34] Adeyemo AA, Bello OO. Preconception care: What women know, think and do. African Journal of Medical and Health Sciences. 2021 Feb 28;20(2):18–26.

[35] Taddese F. Knowledge and Uptake of Preconception Care Among Reproductive Age Women with Chronic Medical Illness Visiting St. Paul’s Millennium Medical College: A Cross sectional Study (Doctoral dissertation).

[36] Setegn M. What Women Do Before Pregnancy A Preconception Care of Women in Mizan Aman town Southwest Ethiopia A Mixed Study. Primary Health Care: Open Access. 2021 Jan 22;11(1):1–6.

[37] Teshome F, Kebede Y, Abamecha F, Birhanu Z Practice of Preconception Care and Associated Factors among Pregnant Women in Manna District, Southwest Ethiopia: A Community-Based Cross-Sectional Study. J Women’s Health Care. 2021 March 8:10(3): 519 10.1136/bmjopen-2019-035937 PMC7380725 32709644

[38] Lawal TA, Adeleye AO. Determinants of folic acid intake during preconception and in early pregnancy by mothers in Ibadan, Nigeria. The Pan African Medical Journal. 2014;19. doi: 10.11604/pamj.2014.19.113.4448 25722786 PMC4337375

[39] Alsammani MA, Kunna A, Adam EM. Factors associated with folic acid knowledge and intake among pregnant women in Sudan. Eastern Mediterranean Health Journal. 2017 Dec 14;23(10):662–9. doi: 10.26719/2017.23.10.662 29270966

[40] Boakye-Yiadom AK, Sagru-Larr ES, ODURO E, ASUMADU OK, SAAH JA, ASARE RO. Preconception care: awareness, knowledge, attitude And practice of pregnant women, tamale west hospital. American Journal Of Health, Medicine And Nursing Practice. 2020 Jun 27;5(1):66–83.

[41] Ahmed K, Saeed A, Alawad A. Knowledge, attitude and practice of preconception care among Sudanese women in reproductive age about rheumatic heart disease. Int J Public Health. 2015 Aug 12;3(5):223–7.

[42] Ezegwui HU, Dim C, Dim N, Ikeme AC. Preconception care in south eastern Nigeria. Journal of Obstetrics and Gynaecology. 2008 Jan 1;28(8):765–8. doi: 10.1080/01443610802462647 19085540

[43] Al Darzi W, Al Mudares F, Farah A, Ali A, Marzouk D. Knowledge of periconceptional folic acid use among pregnant women at Ain Shams University Hospital, Cairo, Egypt. EMHJ-Eastern Mediterranean Health Journal. 2014 Sep 1;20(9):561–8. 25343469

[44] Dessie MA, Zeleke EG, Workie SB, Berihun AW. Folic acid usage and associated factors in the prevention of neural tube defects among pregnant women in Ethiopia: cross-sectional study. BMC pregnancy and childbirth. 2017 Dec;17(1):1–8. 28934941 10.1186/s12884-017-1506-2 PMC5609063

[45] Okon UA, Ibrahim BS, Usman R, Adedire E, Balogun MS, Olayinka A. Awareness and use of folic acid among women of childbearing age in Benue State, Nigeria. The Pan African Medical Journal. 2020:37. doi: 10.11604/pamj.2020.37.60.22848 33209187 PMC7648467

[46] Abdulmalek LJ. Knowledge, attitude and practice regarding folic acid among pregnant women in Benghazi, Libya. Age (years). 2017;20(9/131):7.

[47] Senoga I. Knowledge, attitudes and practices regarding iron-folic acid (IFA) supplementation in pregnancy: A case study of ANC clients attending KCCA health centres, Kampala.

[48] Adebo OO, Dairo DM, Ndikom CM, Adejumo PO. Knowledge and uptake of folic acid among pregnant women attending a secondary health facility in Nigeria. British Journal of Midwifery. 2017 Jun 2;25(6):358–64.

[49] Anzaku AS. Assessing folic acid awareness and its usage for the prevention of neural tube defects among pregnant women in Jos, Nigeria. Journal of Basic and Clinical Reproductive Sciences. 2013;2(1):13–7.

[50] ENUKU C A., Adeyemo FO. Awareness and intake of folic acid by reproductive age women in ozoro, isoko north local government, delta state, Nigeria International Journal of Nursing and Medical Science (IJNMS); 2019; 8 (2); 10–24

[51] Habte A, Dessu S, Haile D. Determinants of practice of preconception care among women of reproductive age group in southern Ethiopia, 2020: content analysis. Reproductive Health. 2021 Dec;18(1):1–4. 34020669 10.1186/s12978-021-01154-3 PMC8139064

[52] Johnson K, Posner SF, Biermann J, Cordero JF, Atrash HK, Parker CS, et al. Recommendations to improve preconception health and Health Care—United States: report of the CDC/ATSDR preconception care work group and the select panel on preconception care. Morbidity and Mortality Weekly Report: Recommendations and Reports. 2006 Apr 21;55(6):1–CE. 16617292

[53] Braspenningx S, Haagdorens M, Blaumeiser B, Jacquemyn Y, Mortier G. Preconceptional care: a systematic review of the current situation and recommendations for the future. Facts, views & vision in ObGyn. 2013;5(1):13. 24753925 PMC3987351

[54] Shamliyan T, Kane RL, Dickinson S. A systematic review of tools used to assess the quality of observational studies that examine incidence or prevalence and risk factors for diseases. Journal of clinical epidemiology. 2010 Oct 1;63(10):1061–70. doi: 10.1016/j.jclinepi.2010.04.014 20728045

[55] Higgins JP. Cochrane handbook for systematic reviews of interventions version 5.0. 1. The Cochrane Collaboration. http://www.cochrane-handbook.org. 2008.

[56] Czeizel AE, Dudas I, Metneki J. Pregnancy outcomes in a randomised controlled trial of periconceptional multivitamin supplementation. Archives of gynecology and obstetrics. 1994 Jul;255(3):131–9. doi: 10.1007/BF02390940 7979565

[57] Kirke PN, Daly LE, Elwood JH. A randomised trial of low dose folic acid to prevent neural tube defects. The Irish Vitamin Study Group. Archives of disease in childhood. 1992 Dec 1;67(12):1442–6. doi: 10.1136/adc.67.12.1442 1489222 PMC1793975

[58] MRC Vitamin Study Research Group. Prevention of neural tube defects: results of the Medical Research Council Vitamin Study. The lancet. 1991 Jul 20;338(8760):131–7. 1677062

[59] Lassi ZS, Kedzior SG, Tariq W, Jadoon Y, Das JK, Bhutta ZA. Effects of preconception care and periconception interventions on maternal nutritional status and birth outcomes in low-and middle-income countries: A systematic review. Nutrients. 2020 Mar;12(3):606. doi: 10.3390/nu12030606 32110886 PMC7146400

[60] Lu MC. Recommendations for preconception care. American Family Physician. 2007 Aug 1;76(3):397–400. 17708141

[61] Dean S, Rudan I, Althabe F, Girard AW, Howson C, Langer A, et al. Setting research priorities for preconception care in low-and middle-income countries: aiming to reduce maternal and child mortality and morbidity. PLoS Med. 2013 Sep 3;10(9):e1001508. doi: 10.1371/journal.pmed.1001508 24019762 PMC3760783

[62] Ayele AD, Belay HG, Kassa BG, Worke MD. Knowledge and utilisation of preconception care and associated factors among women in Ethiopia: systematic review and meta-analysis. Reproductive health. 2021 Dec;18(1):1–5. 33858438 10.1186/s12978-021-01132-9 PMC8048176

[63] Ojifinni OO, Ibisomi L. Exploring the need for preconception care: the pregnancy experiences of women with pre-existing medical conditions in Ibadan, Nigeria. African Journal of Reproductive Health. 2021 May 19;25(2):28–38. 10.29063/ajrh2021/v25i2.3 37585751

