# Supplementary material for: Mothers’ utilization and associated factors of preconception care in Africa, a systematic review and meta-analysis
Source: PLoS One. 2021 Jul 23;16(7):e0254935. doi: 10.1371/journal.pone.0254935 (PMC8301666; doi:10.1371/journal.pone.0254935)
Supplement: Attachment — Submitted filename: Response to reviewers 2.docx [file pone.0254935.s003.docx]

Thank you for all constructive comments

**Response to reviewers**

Journal Requirements:

Please review your reference list to ensure that it is complete and correct. If you have cited papers that have been retracted, please include the rationale for doing so in the manuscript text, or remove these references and replace them with relevant current references. Any changes to the reference list should be mentioned in the rebuttal letter that accompanies your revised manuscript. If you need to cite a retracted article, indicate the article’s retracted status in the References list and also include a citation and full reference for the retraction notice.

**Response – revised**

1. Is the manuscript technically sound, and do the data support the conclusions?

**Response – corrected**

2. Is the manuscript presented in an intelligible fashion and written in standard English?

**Response – corrected**
