# Supplementary material for: Mothers’ utilization and associated factors of preconception care in Africa, a systematic review and meta-analysis
Source: PLoS One. 2021 Jul 23;16(7):e0254935. doi: 10.1371/journal.pone.0254935 (PMC8301666; doi:10.1371/journal.pone.0254935)
Supplement: Attachment — Submitted filename: Response to reviewers 3.docx [file pone.0254935.s004.docx]

Response to reviewers

Thank you for the comments

Journal Requirements:

Please review your reference list to ensure that it is complete and correct. If you have cited papers that have been retracted, please include the rationale for doing so in the manuscript text, or remove these references and replace them with relevant current references. Any changes to the reference list should be mentioned in the rebuttal letter that accompanies your revised manuscript. If you need to cite a retracted article, indicate the article’s retracted status in the References list and also include a citation and full reference for the retraction notice.

**Response -** all references were corrected with the help of google scholar but reference number 9, was cited by the way authors suggestion, due to we are unable to got in google scholar , it is not retracted

- About reference number, 22 , 57 – we used citation suggested in the journal but it differs in google scholar- so we corrected I accordingly

**Review Comments to the Author**

Reviewer #1: Thank you for your revisions and this will be my final comment.

The result presentation in the abstract is not correct.
Please check carefully before publication as this would affect the quality of your paper.

"Knowledge of preconception care (P=0. 61), preexisting medical condition (P=0.71), and
pregnancy intention (p =2.47) were significantly associated with the utilization of preconception
care"

If "P" refers to the p-value, they are not significant. I believe you are referring to the odds ratio.

**Response- corrected**
